# A PUF-Based Secure Authentication and Key Agreement Scheme for the Internet of Drones

**DOI:** 10.3390/s25030982

**Published:** 2025-02-06

**Authors:** Jihye Choi, Seunghwan Son, Deokkyu Kwon, Youngho Park

**Affiliations:** School of Electronic and Electrical Engineering, Kyungpook National University, Daegu 41566, Republic of Korea; jihye@knu.ac.kr (J.C.); sonshawn@knu.ac.kr (S.S.)

**Keywords:** Internet of Drones, PUF, authentication, cryptanalysis, security

## Abstract

The Internet of Drones (IoD) is an emerging industry that offers convenient services for humans due to the high mobility and flexibility of drones. The IoD substantially enhances human life by enabling diverse drone applications across various domains. However, a malicious adversary can attempt security attacks because communication within an IoD environment is conducted through public channels and because drones are vulnerable to physical attacks. In 2023, Sharma et al. proposed a physical unclonable function (PUF)-based authentication and key agreement (AKA) scheme for the IoD. Regrettably, we discover that their scheme cannot prevent impersonation, stolen verifier, and ephemeral secret leakage (ESL) attacks. Moreover, Sharma et al.’s scheme cannot preserve user untraceability and anonymity. In this paper, we propose a secure and lightweight AKA scheme which addresses the shortcomings of Sharma et al.’s scheme. The proposed scheme has resistance against diverse security attacks, including physical capture attacks on drones, by leveraging a PUF. Furthermore, we utilize lightweight operations such as hash function and XOR operation to accommodate the computational constraints of drones. The security of the proposed scheme is rigorously verified, utilizing “Burrows–Abadi–Needham (BAN) logic”, “Real-or-Random (ROR) model”, “Automated Validation of Internet Security Protocols and Application (AVISPA)”, and informal analysis. Additionally, we compare the security properties, computational cost, communication cost, and energy consumption of the proposed scheme with other related works to evaluate performance. As a result, we determine that our scheme is efficient and well suited for the IoD.

## 1. Introduction

The “Internet of Drones (IoD)” [1] is considered as a prominent industry that is shaping the future of human life through the diverse applications and capabilities of drones. With their high mobility and flexibility, drones are ideally suited for performing tasks across various domains [2]. Drones provide effective alternatives for performing tasks that are labor-intensive or challenging for human operators. The IoD is a network architecture which coordinates drone access and manages their operations within the Internet [3]. Generally, the IoD architecture consists of a control station server (CSS), drones, and remote users. The CSS acts as the control center, overseeing drone operations to ensure appropriate functionality and facilitating communication between drones and remote users. Drones are equipped with various sensors, computational capabilities, and communication modules and can connect to a CSS via the Internet to execute a range of tasks [4]. Drones can be deployed in various environments and provide a wide range of services, including traffic monitoring, aerial photography, delivery, rescue, and surveillance [5]. Drones collect the surrounding data and transmit them to a CSS or share it with remote users through CSS arbitration [6]. This interconnected structure enables drones to offer convenient services to remote users who benefit from enhanced functionality.

Although the IoD presents various advantages for enhancing human life, it still encounters several critical challenges requiring resolution. In the IoD architecture, communication between drones, remote users, and the CSS occurs through public channels [7]. This exposes the IoD system to potential attacks comprising replay, eavesdropping, insider, and man-in-the-middle (MITM) attacks [8,9]. Additionally, drones are susceptible to unauthorized physical access as they operate in open airspace [10]. A malicious attacker can hijack or physically capture a drone to obtain sensitive data and attempt to disrupt drone operation by injecting malicious payloads. Such breaches can compromise user privacy and lead to substantial security risks. To address these vulnerabilities, various security technologies have been proposed for IoD environments, such as intrusion detection system and anti-jamming [11,12,13]. In this paper, we focus on the authentication and key agreement (AKA) to preserve privacy, determinate identity of network participants, and establish secure communication channels between users and drones. Another pressing challenge is lightweight computation for drones. Drones have limitations of processing capabilities and database capacity [14], which makes them differ from a CSS, which operates in environments with abundant computing power and storage. Computations are completed within a constrained timeframe to eliminate time delay as the IoD services rely on real-time operation. As a result, it is indispensable to design a secure and lightweight AKA scheme for the IoD in order to guarantee efficient performance while maintaining data security and computational efficiency.

In recent years, various AKA schemes have been proposed to provide security for IoD environments [15,16,17,18]. However, such schemes suffer from challenges in lightweight operation and resistance to security vulnerabilities, including physical attacks, which are important issues in IoD environments. To overcome these vulnerabilities, Sharma et al. [19] proposed a physical unclonable function (PUF)-based AKA scheme for the IoD in 2023. Their scheme considered the computational limitations of drones by employing the hash function, exclusive-OR (XOR), and PUF. Sharma et al. argued that their scheme defends numerous adversarial attacks, including privileged insider, MITM, replay, and drone capture attacks. Unfortunately, we demonstrate that their scheme cannot prevent impersonation, stolen verifier, and ephemeral secret leakage (ESL) attacks. Specifically, the session key shared by the user and the drone is exposed by an adversary, compromising mutual authentication. Furthermore, their scheme fails to guarantee user untraceability and anonymity. Therefore, we propose a robust and secure AKA scheme that addresses the flaws of Sharma et al.’s scheme. The proposed scheme defends diverse attacks containing drone capture, impersonation, stolen verifier, and ESL attacks. Moreover, the proposed scheme adopts a PUF that is similar to the approach utilized in Sharma et al.’s scheme. Drones can generate a secret key masked with “challenge–response” pair and protect the data stored in their memory using the key. The proposed scheme achieves enhanced security mitigating the security shortcomings of Sharma et al.’s scheme. Our scheme effectively prevents various security threats including impersonation, stolen verifier, and ESL attacks while introducing additional security properties. Moreover, the proposed scheme achieves a better balance between security and cost efficiency. Compared to Sharma et al.’s scheme, our scheme offers improved security without compromising performance or practicality.

### 1.1. Contributions

This study offers the following major contributions:

- We analyze Sharma et al.’s scheme and indicate the security weaknesses related to impersonation, stolen verifier, and ESL attacks of their scheme. Furthermore, we demonstrate that their scheme does not guarantee mutual authentication, user untraceability, and anonymity.
- We suggest a lightweight and secure AKA scheme to mitigate the drawbacks of Sharma et al.’s scheme. The proposed scheme adopts one-way hash functions and XOR operations, which are suitable for drones with limited computing power. Additionally, we incorporate a PUF to manage the data stored in drones securely and prevent unauthorized accesses to drones.
- We demonstrate that our scheme ensures the robustness against numerous attacks by performing informal analysis. Moreover, we conduct “Burrows–Abadi–Needham (BAN) logic”, “Real-or-Random (ROR) model”, and “Automated Validation of Internet Security Protocols and Application (AVISPA)”, which represent the resilience of our scheme formally.
- We prove that our scheme achieves cost efficiency with respect to computational cost, communication cost, and energy consumption by conducting a comparison between the proposed scheme and other relevant schemes.

### 1.2. Organization

We discuss associated studies for the IoD in Section 2. We provide an explanation of the IoD architecture model, adversary model, and the properties of a PUF in Section 3. We revisit Sharma et al.’s scheme in Section 4. We conduct a cryptanalysis of Sharma et al.’s scheme to verify that their scheme has security vulnerabilities in Section 5. We propose a secure and cost-effective AKA scheme for the IoD, which remedies the flaws identified in Sharma et al.’s scheme in Section 6. We assess the resilience of the proposed AKA scheme by adopting various examination methods in Section 7. We highlight the robustness and efficiency through a comparative analysis between the proposed and relevant schemes in Section 8. Finally, we wrap up our study with concluding remarks in Section 9.

## 2. Related Works

The IoD is a rapidly growing industry that attracts significant attention, prompting researchers to develop AKA schemes for secure IoD communication. In 2021, Nikooghadam et al. [20] devised an AKA scheme for smart city surveillance to construct secure communication between user and drone. They used elliptic curve cryptography (ECC) to enhance energy costs more than traditional public-key cryptosystems (e.g., RSA). Unfortunately, Alzahrani et al. [21] indicated that Nikooghadam et al.’s scheme cannot defend stolen verifier and insider attacks, and that it also lacks user anonymity and untraceability. They proposed an AKA scheme between a user and drone that addresses the security vulnerabilities of Nikooghadam et al.’s scheme. However, their scheme still suffers from security attacks, including drone capture and insider attacks, and cannot ensure security properties, including user anonymity, message integrity, and confidentiality [22]. Tanveer et al. [23] presented an AKA protocol for the IoD environment using ECC. They utilized AEGIS and ECC to enhance their scheme. However, the scheme cannot prevent impersonation and drone capture attacks [24]. Dwivedi et al. [25] propounded a data delivery AKA scheme for tactile Internet-enabled IoD. Their scheme employs ECC and blockchain, providing security for various attacks. It also provides user anonymity, unlinkability, and data immutability. However, previously proposed schemes [20,21,23,25] use ECC, which involves high-complexity computation unsuitable for drones. Because drones are constraint with regard to their computing power, a lightweight authentication protocol is required for the IoD.

Therefore, many researchers have focused on designing protocols with lightweight computational overhead. Ali et al. [26] devised a biometric-based AKA scheme between user and drone for smart city surveillance. Their scheme used lightweight operations such as hash function, XOR operation, and symmetric encryption. Regrettably, the scheme has weaknesses related to server session key disclosure, spoofing, and forgery attacks [27]. Chaudhary et al. [28] designed an anonymous AKA scheme for the IoD. Their scheme uses only an XOR operation and a one-way hash function for computational efficiency. Unfortunately, their scheme is vulnerable to user impersonation attacks and cannot preserve user privacy protection. Lee et al. [29] propounded a lightweight AKA protocol for the IoD using a one-way hash function and an XOR operation. Although they assert that their scheme rectifies the vulnerabilities of Chaudhary et al.’s scheme and is resistant against numerous attacks, it is still susceptible to the physical attacks of drones. Hussain et al. [30] also presented a lightweight authentication protocol for the IoD environment using symmetric encryption, a one-way hash function, and an XOR operation. The analysis of their scheme shows that it can prevent various attacks. However, it cannot defend against impersonation attacks and physical attacks on drones. Pratap et al. [15] suggested an AKA scheme between a user and a drone for the IoD that addresses the resource limitation issue of drones by utilizing hyperelliptic curve cryptography (HECC). Unfortunately, their scheme is susceptible to drone capture attacks. Although all of these schemes [15,26,28,29,30] are computationally efficient, they exhibit security drawbacks, particularly a susceptibility to drone capture attacks.

To mitigate the risk of physical attack on drones, numerous researchers have carried out studies. Zhang et al. [16] propounded a key management scheme for the IoD. They considered restricted computing power and physical security issue of drones using a PUF and lightweight operations. Tanveer et al. [17] proposed a biometric-based AKA scheme securing information within the IoD infrastructure. They adopted a PUF, a hash function and symmetric encryption to provide secure communication between users and drones. Tanveer et al. [18] also devised a PUF-based authentication scheme, establishing a session key between users and drones. Using a PUF, a hash function, and AEGIS, their scheme addresses the susceptibility and resource constraints of drone communication. Sharma et al. [19] suggested a lightweight and physical attack-resistant AKA scheme for the IoD environment. Regrettably, we identified that Sharma et al.’s has limitations in defending user impersonation, stolen verifier, and ESL attacks. Moreover, user anonymity and untracability are not preserved in their scheme. Therefore, we propose a robust and lightweight AKA scheme to address the shortcomings in Sharma et al.’s scheme. Table 1 represents the summary of the related schemes.

## 3. Preliminaries

In this part, we explain essential concepts and background for a comprehensive understanding of the proposed scheme. We describe the system model, adversary model and the PUF.

### 3.1. System Model

**Figure 1:**
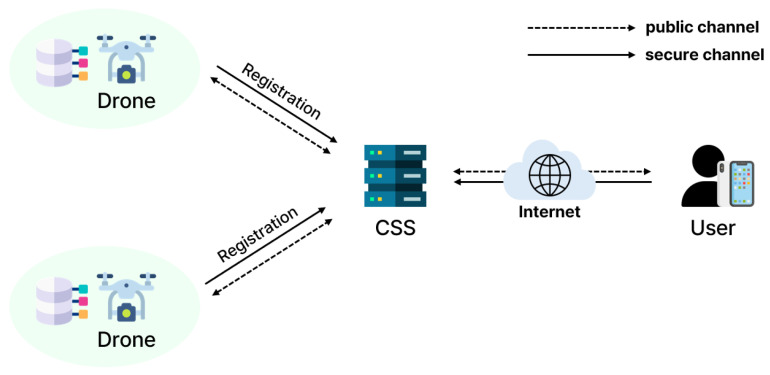
System model for the IoD.

Figure 1 illustrates the IoD architecture. There are three entities in the proposed system model: control station server (CSS), remote users, and drones. These entities communicate through wireless channels.

- The CSS is a fully trusted entity. The CSS possesses abundant resources and extensive memory capabilities for controlling system networks. First, the CSS initializes the entire system and registers users and drones. Sensitive data related to users and drones and the information collected by drones are stored in its database. Users and drones authenticate with the mediation of the CSS.
- Remote users need to authenticate with the CSS to access the data stored in the CSS and utilize convenient services. After mutual authentication support from the CSS, users also can directly access the real-time information gathered by drones.
- Drones are deployed in open airspace and gather surrounding information. The information collected by drones is transmitted to the CSS for further processing. PUFs which are embedded in drones protect the secret parameters stored in drones. If a drone is captured, the PUF will be unusable and authentication with the user or the CSS cannot be completed. Additionally, drones have limited resources and memory capabilities.

### 3.2. Adversary Model

In this paper, we evaluate the security of AKA scheme by adopting the widely operated threat models “Dolev-Yao (DY)” [31] and “Canetti-Krawczyk (CK)” [32]. The DY and CK models provide the assumptions used to characterize the potential of an adversary. A malicious adversary A can delete, insert, eavesdrop, revise, and re-transmit messages sent through a public channel. Moreover, A can obtain and expose session state and temporary session keys or the master key of the CSS. Based on following assumptions, we assess the security of the proposed scheme.

- A can steal a smart device of a remote user and and use power analysis attacks to retrieve secret credentials stored in the device [33,34].
- A can be a legitimate user of the system or an outsider and can attempt various attacks using obtained information.
- A can steal the verification table stored in the CSS and can attempt various attacks using obtained information.
- A can attempt a variety of attacks, including MITM, privileged insider, replay, and impersonation attacks.

### 3.3. Physical Unclonable Function

The microstructure of the hardware exhibits unique physical deviations generated by manufacturing disparities. The PUF depends on the characteristic property of the microstructure. The PUF can be considered as fingerprint of the hardware. A PUF includes a unique input–output pair called the “challenge–response” pair. We can use a unique response for authentication and key generation. In this paper, we illustrate the operation of a PUF as R=PUF(C). The notation *C* indicates a challenge and *R* indicates a response. We describe the attributes of the PUF as follows:

- A PUF is an unclonable circuit. It is impossible for any ${PUF}^{\prime }\left(\cdot \right)$ to satisfy ${PUF}^{\prime }\left(C\right)=PUF\left(C\right)$.
- While PUF(C)=R can be computed easily, determining *R* for a given *C* within polynomial time is computationally infeasible.
- The output of a PUF is unpredictable [35].

In the proposed scheme, we adopt a PUF to prevent unauthorized physical accesses on drones and protect secret information stored in their memory. Drones can use PUF responses as a secret key using its uniqueness.

## 4. Review of Sharma et al.’s Scheme

An overview of Sharma et al.’s scheme is provided here. Table 2 summarizes the key notations utilized in Sharma et al.’s scheme. The following outlines its details:

### 4.1. Initialization Phase

Initially, the CSS chooses its identity $ID_{CSS}$, a secret key $X_{CSS}$, and a one-way hash function h(·). Then, the CSS calculates a pseudo-identity $CID_{CSS}=h(X_{CSS}||ID_{CSS})$ and publishes h(·) and $CID_{CSS}$.

### 4.2. Drone Registration Phase

**Step** **1:** $D_{j}$ picks its identity $DID_{j}$ and a challenge *C*, and computes R=PUF(C). Then, $D_{j}$ sends $\left\{DID_{j},C,R\right\}$ to the CSS through a secure channel.
**Step** **2:** The CSS calculates $PDID_{j}=h(DID_{j}\left|\right|X_{CSS})$ after receiving the message and stores $\left\{PDID_{j},C,R\right\}$ in the database. Then, the CSS transmits $\left\{PDID_{j}\right\}$ to $D_{j}$ securely.
**Step** **3:** $D_{j}$ saves $\left\{PDID_{j},C,CID_{CSS}\right\}$ to a database.

### 4.3. User Registration Phase

**Step** **1:** $U_{i}$ selects $ID_{i}$ and $PW_{i}$. Then, $U_{i}$ transmits $ID_{i}$ to the CSS securely.
**Step** **2:** The CSS computes $PID_{i}=h(ID_{i}\left|\right|X_{CSS})$ and $s_{i}=h(PID_{i}\left|\right|X_{CSS})$ upon receiving the message. The CSS sends $\left\{PID_{i},s_{i},PDID_{j},C\right\}$ to $U_{i}$ through a secure channel after storing $\left\{ID_{i},PID_{i},s_{i}\right\}$ in the database.
**Step** **3:** $U_{i}$ calculates $s_{i}^{\prime }=s_{i}⊕h(ID_{i}||PW_{i})$ and $PID_{i}^{\prime }=PID_{i}⊕h(ID_{i}||PW_{i})$. Finally, $U_{i}$ stores $\left\{s_{i}^{\prime },PID_{i}^{\prime },C,PDID_{j}\right\}$.

### 4.4. Authentication and Key Agreement Phase

**Figure 2:**
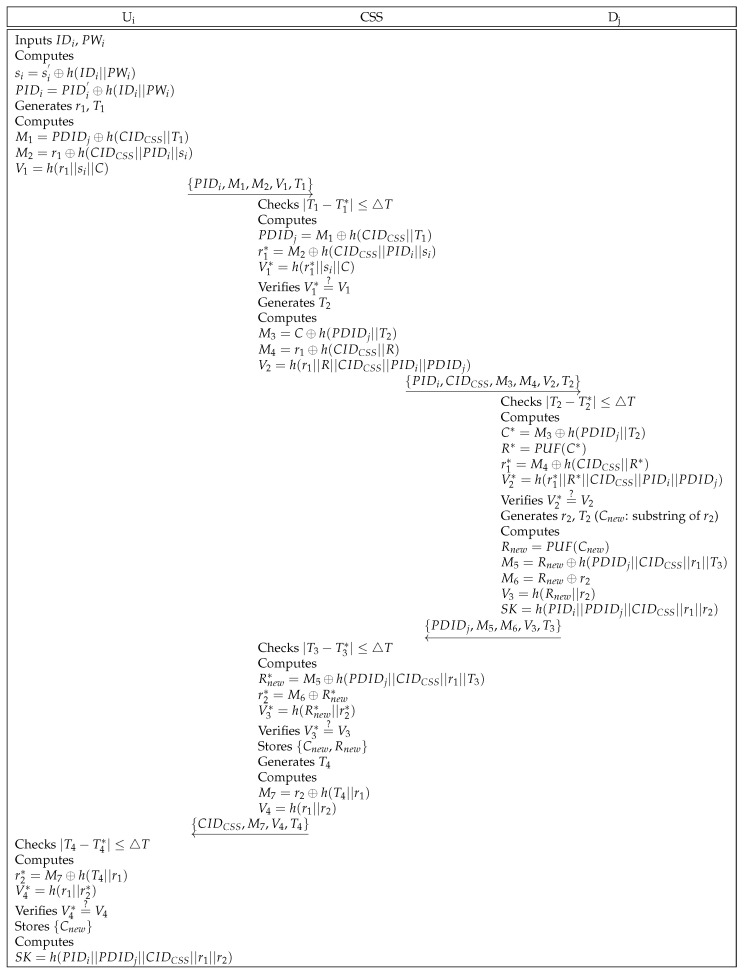
Authentication and key agreement phase of Sharma et al.’s scheme.

First, the user $U_{i}$ transmits an authentication request message to the CSS. The CSS mediates between the user $U_{i}$ and the drone $D_{j}$, verifying whether $U_{i}$ and $D_{j}$ are legitimate or not. Finally, $U_{i}$ and $D_{j}$ share a session key for establishing secure communication. Figure 2 indicates the processes of authentication and key agreement.

**Step** **1:** $U_{i}$ inserts identity $ID_{i}$ and password $PW_{i}$, and computes $s_{i}=s_{i}^{\prime }⊕h(ID_{i}||PW_{i})$, and $PID_{i}=PID_{i}^{\prime }⊕h(ID_{i}||PW_{i})$. Then, $U_{i}$ generates a random number $r_{1}$ and timestamp $T_{1}$ and calculates $M_{1}=PDID_{j}⊕h(CID_{CSS}\left|\right|T_{1})$, $M_{2}=r_{1}⊕h(CID_{CSS}||PID_{i}\left|\right|s_{i})$, and $V_{1}=h(r_{1}\left|\right|s_{i}\left||C\right)$. Further, $U_{i}$ sends the message $\left\{PID_{i},M_{1},M_{2},V_{1},T_{1}\right\}$ to the CSS through an open channel.
**Step** **2:** The CSS first checks whether $T_{1}$ is valid or not. If it is valid, the CSS retrieves $s_{i}$ against $PID_{i}$ and computes $PDID_{j}=M_{1}⊕h(CID_{CSS}\left|\right|T_{1})$, $r_{1}^{*}=M_{2}⊕h(CID_{CSS}||PID_{i}\left|\right|s_{i})$, and $V_{1}^{*}=h(r_{1}^{*}\left|\right|s_{i}\left||C\right)$. Then, the CSS verifies that $V_{1}^{*}$ is equal to $V_{1}$. If they are identical, the CSS generates a timestamp $T_{2}$, and calculates $M_{3}=C⊕h(PDID_{j}\left|\right|T_{2})$, $M_{4}=r_{1}⊕h(CID_{CSS}\left||R\right)$, and $V_{2}=h(r_{1}||R||CID_{CSS}||PID_{i}||PDID_{j})$. The CSS transmits $\{PID_{i},CID_{CSS},$$M_{3},M_{4},V_{2},T_{2}\}$ over a public channel.
**Step** **3:** $D_{j}$ verifies the legitimacy of $T_{2}$. If it is legitimate, $D_{j}$ computes $C^{*}=M_{3}⊕h(PDID_{j}\left|\right|T_{2})$, $R^{*}=PUF\left(C^{*}\right)$, $r_{1}^{*}=M_{4}⊕h(CID_{CSS}\left|\right|R^{*})$, and $V_{2}^{*}=h(r_{1}^{*}\left|\right|R^{*}||CID_{CSS}\left|\right|$$PID_{i}||PDID_{j})$. Then, $D_{j}$ checks whether $V_{2}^{*}$ and $V_{2}$ are equal or not. After checking the equality, $D_{j}$ generates a random number $r_{2}$ and a timestamp $T_{3}$. $C_{new}$ is a substring of $r_{2}$. After that, $D_{j}$ calculates $R_{new}=PUF\left(C_{new}\right)$, $M_{5}=R_{new}⊕h(PDID_{j}||CID_{CSS}\left|\right|r_{1}\left|\right|T_{3})$, $M_{6}=R_{new}⊕r_{2}$, $V_{3}=h(R_{new}\left|\right|r_{2})$, and $SK=h(PID_{i}||PDID_{j}$$||CID_{CSS}\left|\right|r_{1}\left|\right|r_{2})$, and sends $\left\{PDID_{j},M_{5},M_{6},V_{3},T_{3}\right\}$ to the CSS through a public channel.
**Step** **4:** The CSS checks the validity of $T_{3}$. If it is valid, the CSS calculates $R_{new}^{*}=M_{5}⊕h(PDID_{j}\left|\right|$$CID_{CSS}\left|\right|r_{1}\left|\right|T_{3})$, $r_{2}^{*}=M_{6}⊕R_{new}^{*}$, and $V_{3}^{*}=h(R_{new}^{*}\left|\right|r_{2}^{*})$. Further, the CSS compares $V_{4}^{*}$ with $V_{4}$. If they are equal, the CSS stores $\left\{C_{new},R_{new}\right\}$ in the database and generates a timestamp $T_{4}$. The CSS computes $M_{7}=r_{2}⊕h(T_{4}\left|\right|r_{1})$ and $V_{4}=h(r_{1}\left|\right|r_{2})$, and transmits $\left\{CID_{CSS},M_{7},V_{4},T_{4}\right\}$ to $U_{i}$.
**Step** **5:** $U_{i}$ verifies that $T_{4}$ is legitimate. If legitimate, $U_{i}$ computes $r_{2}^{*}=M_{7}⊕h(T_{4}\left|\right|r_{1})$ and $V_{4}^{*}=h(r_{1}\left|\right|r_{2}^{*})$. Then, $U_{i}$ checks that $V_{4}^{*}$ is equal to $V_{4}$. If they are equal, $U_{i}$ stores $C_{new}$ and establishes the session key $SK=h(PID_{i}||PDID_{j}||CID_{CSS}||r_{1}||r_{2})$.

## 5. Cryptanalysis of Sharma et al.’s Scheme

Cryptanalysis is conducted to indicate that Sharma et al.’s scheme cannot prevent impersonation, stolen verifier, ESL attacks and cannot ensure user anonymity and untraceability. The detailed steps are outlined as follows:

### 5.1. User Impersonation Attack

A malicious adversary A impersonates a legitimate user using the secret parameters extracted from user’s smart device. Then, A establishes a session key with a drone. The details are outlined below.

**Step** **1:** A can exploit a power analysis attack to extract the secret information $\{s_{i}^{\prime },PID_{i}^{\prime },C,$$PDID_{j}\}$ stored on the user’s smart device, under the assumptions described in Section 3.2.
**Step** **2:** A eavesdrops on $PID_{i}$ transmitted through a public channel and obtains $h(ID_{i}||PW_{i})=PID_{i}^{\prime }⊕PID_{i}$. Then, A can calculate $s_{i}=s_{i}^{\prime }⊕h(ID_{i}||PW_{i})$.
**Step** **3:** A generates a number $r_{A}$ randomly and a timestamp $T_{A}$, and calculates the request messages $M_{1}=PDID_{j}⊕h(CID_{CSS}\left|\right|T_{A})$, $M_{2}=r_{A}⊕h(CID_{CSS}||PID_{i}\left|\right|s_{i})$, and $V_{1}=h(r_{A}\left|\right|s_{i}\left||C\right)$.
**Step** **4:** The CSS receives the request message and delivers the random number of A to $D_{j}$. Then, $D_{j}$ computes a session key $SK=h(PID_{i}||PDID_{j}||CID_{CSS}||r_{A}||r_{2})$ and transmits $\left\{PDID_{j},M_{5},M_{6},V_{3},T_{3}\right\}$ to the CSS.
**Step** **5:** The CSS authenticates $D_{j}$ and sends the message $M_{7}=r_{2}⊕h(T_{4}\left|\right|r_{A})$ to A. Finally, A obtains $r_{2}=M_{7}⊕h(T_{4}\left|\right|r_{1})$ and computes $SK=h(PID_{i}||PDID_{j}||CID_{CSS}||r_{A}||r_{2})$.

### 5.2. Stolen Verifier Attack

Under the CK model, A can access the verification table $\left\{ID_{i},PID_{i},s_{i}\right\}$ stored in the database of the CSS. Further, A can access the pseudo-identities of each of $\{PID_{i},PDID_{j},$ and $CID_{CSS}\}$ entities, because they are transmitted through an open channel and not updated. To compute the session key between $U_{i}$ and $D_{j}$, A calculates $r_{1}=M_{2}⊕h(CID_{CSS}||PID_{i}\left|s_{i}\right)$ and $r_{2}=M_{7}⊕h(T_{4}\left|\right|r_{1})$, where $M_{2}$ and $M_{7}$ are sent through an open channel. Finally, A can obtain the session key $SK=h(PID_{i}||PDID_{j}||CID_{CSS}||r_{1}||r_{2})$.

### 5.3. Ephemeral Secret Leakage Attack

In Sharma et al.’s scheme, $U_{i}$ and $D_{j}$ establish a session key using the pseudo-identities of each $\left\{PID_{i},PDID_{j},CID_{CSS}\right\}$ entity and the random numbers $\left\{r_{1},r_{2}\right\}$ generated by $U_{i}$ and $D_{j}$. Therefore, if A gains those values, A can calculate the session key shared between $U_{i}$ and $D_{j}$. Under the CK model, A can acquire the ephemeral random numbers $r_{1},r_{2}$ generated during a session. Furthermore, A can eavesdrop on the pseudo-identities $\left\{PID_{i},PDID_{j},CID_{CSS}\right\}$ sent through an open channel. As a result, A can derive the session key $SK=h(PID_{i}||PDID_{j}||CID_{CSS}||r_{1}||r_{2})$.

### 5.4. User Anonymity and Untraceability

A can eavesdrop the message sent through a public channel in accordance with the adversary model described in Section 3.2. In the AKA phase of Sharma et al.’s scheme, $U_{i}$ and the CSS transmit $PID_{i}$ through a public channel. At the end of the AKA phase, they do not update $PID_{i}$. Therefore, Sharma et al.’s scheme lacks the ability to preserve user untraceability and anonymity.

## 6. Proposed Scheme

Here, we detail our AKA scheme for the IoD, designed with PUF technology. The proposed scheme comprises the following phases: (1) initialization, (2) registration, (3) authentication and key agreement, and (4) password update. Users and drones register themselves to the CSS and share a session key with arbitration of the CSS. Detailed steps are outlined as follows.

### 6.1. Initialization

The CSS selects h(·) as a one-way hash function, along with a secret key $X_{CSS}$ and an identity $CID_{CSS}$. Then, the CSS publishes h(·).

### 6.2. Drone Registration Phase

**Figure 3:**
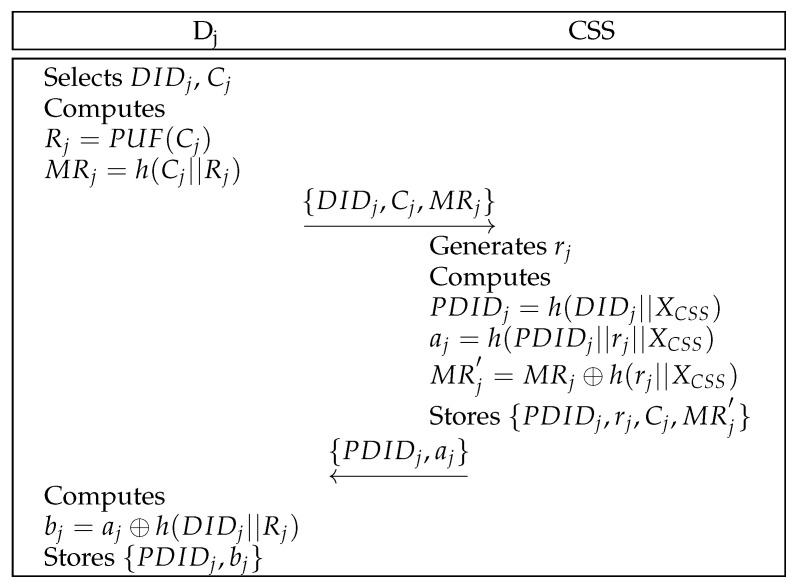
Drone registration of the proposed scheme.

A drone registers itself with the CSS before authentication. Figure 3 represents the procedures of drone registration. Details are outlined below.

**Step** **1:** $D_{j}$ chooses its identity $DID_{j}$ and a challenge *C*, and computes R=PUF(C) and $MR_{j}=h(C_{j}\left|\right|R_{j})$. Then, $D_{j}$ sends $\left\{DID_{j},MR_{j}\right\}$ to the CSS securely.
**Step** **2:** The CSS generates a random number $r_{j}$, and calculates $PDID_{j}=h(DID_{j}\left|\right|X_{CSS})$, $a_{j}=h(PDID_{j}\left|\right|r_{j}\left|\right|X_{CSS})$, and $MR_{j}^{\prime }=MR_{j}⊕h(r_{j}\left|\right|X_{CSS})$ after receiving the message. Then, the CSS stores $\left\{PDID_{j},r_{j},C_{j},MR_{j}^{\prime }\right\}$ in a database and transmits $\left\{PDID_{j},a_{j}\right\}$ to $D_{j}$ securely.
**Step** **3:** $D_{j}$ computes $b_{j}=a_{j}⊕h(DID_{j}\left|\right|R_{j})$, and saves $\left\{PDID_{j},b_{j}\right\}$ to a database.

### 6.3. User Registration Phase

**Figure 4:**
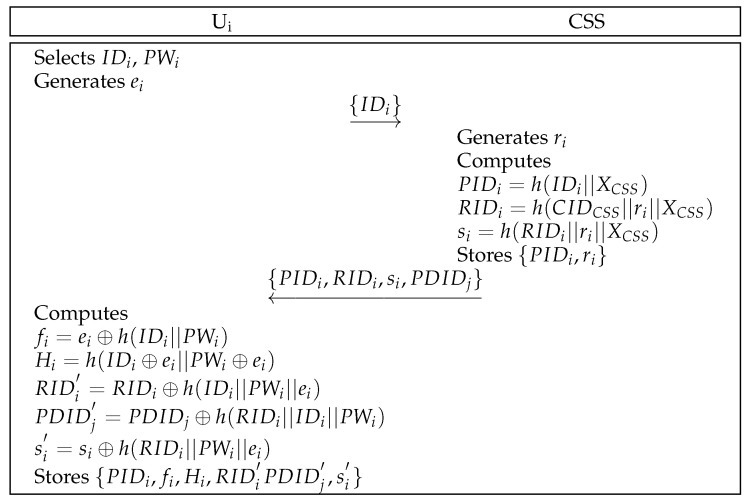
User registration of the proposed scheme.

A user registers themselves with the CSS before authentication. Figure 4 shows the comprehensive steps of user registration. The following steps outline the details of this process.

**Step** **1:** First, $U_{i}$ selects an identity $ID_{i}$ and a password $PW_{i}$. Further, $U_{i}$ generates a number $e_{i}$ randomly and transmits $ID_{i}$ to the CSS securely.
**Step** **2:** Upon receiving the message, the CSS generates a number $r_{i}$ randomly and calculates $PID_{i}=h(ID_{i}\left|\right|X_{CSS})$, $RID_{i}=h(CID_{CSS}\left|\right|r_{i}\left|\right|X_{CSS})$, and $s_{i}=h(PID_{i}\left|\right|X_{CSS})$. The CSS sends $\left\{PID_{i},RID_{i},s_{i},PDID_{j}\right\}$ to $U_{i}$ through secure channel after it stores $\left\{PID_{i},r_{i}\right\}$ in the database.
**Step** **3:** $U_{i}$ calculates $f_{i}=e_{i}⊕h(ID_{i}||PW_{i})$, $H_{i}=h(ID_{i}⊕e_{i}||PW_{i}⊕e_{i})$, $RID_{i}^{\prime }=RID_{i}⊕h(ID_{i}||PW_{i}\left|\right|e_{i})$, $PDID_{j}^{\prime }=PDID_{j}⊕h(RID_{i}||ID_{i}||PW_{i})$, and $s_{i}^{\prime }=s_{i}⊕h(RID_{i}||PW_{i}\left|\right|e_{i})$. Finally, $U_{i}$ stores $\left\{PID_{i},f_{i},H_{i},RID_{i}^{\prime },PDID_{j}^{\prime },s_{i}^{\prime }\right\}$ in the database.

### 6.4. Authentication and Key Agreement Phase

**Figure 5:**
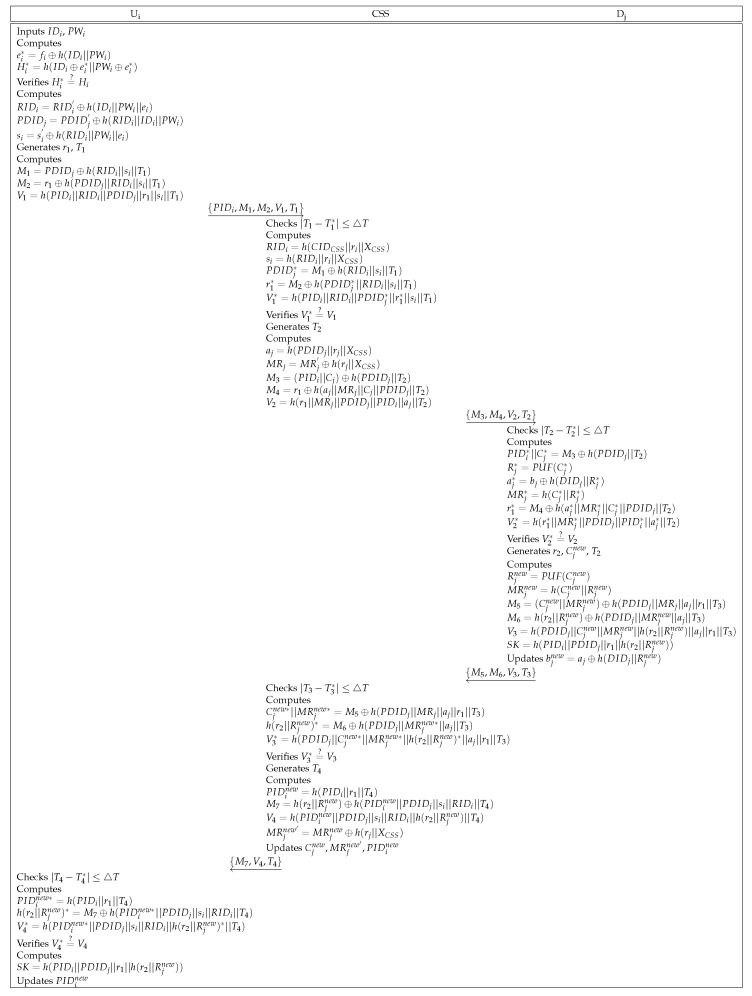
Authentication and key agreement phase of the proposed scheme.

Authentication between $U_{i}$ and $D_{j}$ is established in this phase. After the authentication, they share a session key with the mediation of the CSS. Figure 5 depicts the details of the AKA phase.

**Step** **1:** $U_{i}$ inserts his/her identity $ID_{i}$ and password $PW_{i}$, and computes $e_{i}^{*}=f_{i}⊕h(ID_{i}||PW_{i})$ and $H_{i}^{*}=h(ID_{i}⊕e_{i}^{*}||PW_{i}⊕e_{i}^{*})$. Then, $U_{i}$ compares whether $H_{i}^{*}$ and $H_{i}$ are equal or not. If they are equal, login is completed. $U_{i}$ calculates $RID_{i}=RID_{i}^{\prime }⊕h(ID_{i}||PW_{i}\left|\right|e_{i})$, $PDID_{j}=PDID_{j}^{\prime }⊕h(RID_{i}||ID_{i}||PW_{i})$, and $s_{i}=s_{i}^{\prime }⊕h(RID_{i}||PW_{i}\left|\right|e_{i})$. Then, $U_{i}$ selects a random number $r_{1}$ and a timestamp $T_{1}$, and calculates $M_{1}=PDID_{j}⊕h(RID_{i}\left|\right|s_{i}\left|\right|T_{1})$, $M_{2}=r_{1}⊕h(PDID_{j}||RID_{i}\left|\right|s_{i}\left|\right|T_{1})$, and $V_{1}=h(PID_{i}||RID_{i}\left|\right|$$PDID_{j}\left|\right|r_{1}\left|\right|s_{i}\left|\right|T_{1})$. Further, $U_{i}$ sends a message $\left\{PID_{i},M_{1},M_{2},V_{1},T_{1}\right\}$ to the CSS through an open channel.
**Step** **2:** The CSS first checks whether $T_{1}$ is valid or not. If it is valid, the CSS retrieves $r_{i}$ against $PID_{i}$ and computes $RID_{i}=h(CID_{CSS}\left|\right|r_{i}\left|\right|X_{CSS})$, $s_{i}=h(RID_{i}\left|\right|r_{i}\left|\right|X_{CSS})$, $PDID_{j}^{*}=M_{1}⊕h(RID_{i}\left|\right|s_{i}\left|\right|T_{1})$, $r_{1}^{*}=M_{2}⊕h(PDID_{j}^{*}||RID_{i}\left|\right|s_{i}\left|\right|T_{1})$, and $V_{1}^{*}=h(PID_{i}\left|\right|RID_{i}$$||PDID_{j}^{*}\left|\right|r_{1}^{*}\left|\right|s_{i}\left|\right|T_{1})$. Then, the CSS verifies that $V_{1}^{*}$ is equal to $V_{1}$. If they are equal, the CSS generates a timestamp $T_{2}$ and retrieves $r_{j}$ against $PDID_{j}$. Then, the CSS calculates $a_{j}=h(PDID_{j}\left|\right|r_{j}\left|\right|X_{CSS})$, $MR_{j}=MR_{j}^{\prime }⊕h(r_{j}\left|\right|X_{CSS})$, $M_{3}=(PID_{i}\left|\right|C_{j})⊕h(PDID_{j}\left|\right|T_{2})$, $M_{4}=r_{1}⊕h(a_{j}||MR_{j}\left|\right|C_{j}||PDID_{j}\left|\right|T_{2})$, and $V_{2}=h(r_{1}||MR_{j}||PDID_{j}||PID_{i}\left|\right|a_{j}\left|T_{2}\right)$. The CSS transmits $\left\{M_{3},M_{4},V_{2},T_{2}\right\}$ over a public channel.
**Step** **3:** $D_{j}$ verifies the legitimacy of $T_{2}$. If it is legitimate, $D_{j}$ computes $PID_{i}^{*}\left|\right|C_{j}^{*}=M_{3}⊕h(PDID_{j}\left|\right|T_{2})$, $R_{j}^{*}=PUF\left(C_{j}^{*}\right)$, $a_{j}^{*}=b_{j}⊕h(DID_{j}\left|\right|R_{j}^{*})$, $MR_{j}^{*}=h(C_{j}^{*}\left|\right|R_{j}^{*})$, $r_{1}^{*}=M_{4}⊕h(a_{j}^{*}||MR_{j}^{*}\left|\right|C_{j}^{*}||PDID_{j}\left|\right|T_{2})$, and $V_{2}^{*}=h(r_{1}^{*}||MR_{j}^{*}||PDID_{j}||PID_{i}^{*}\left|\right|a_{j}^{*}\left|\right|T_{2})$. Then, $D_{j}$ checks whether $V_{2}^{*}$ and $V_{2}$ are equal or not. If they are equal, $D_{j}$ generates a random number $r_{2}$, a new challenge $C_{j}^{new}$ and a timestamp $T_{3}$. After that, $D_{j}$ calculates $R_{j}^{new}=PUF\left(C_{j}^{new}\right)$, $MR_{j}^{new}=h(C_{j}^{new}\left|\right|R_{j}^{new})$, $M_{5}=(C_{j}^{new}||MR_{j}^{new})⊕h(PDID_{j}||MR_{j}\left|\right|a_{j}\left|\right|r_{1}\left|\right|T_{3})$, $M_{6}=h(r_{2}\left|\right|R_{j}^{new})⊕h(PDID_{j}||MR_{j}^{new}\left|\right|a_{j}\left|\right|T_{3})$, $V_{3}=h($$PDID_{j}\left|\right|C_{j}^{new}||MR_{j}^{new}||h(r_{2}\left|\right|R_{j}^{new})||a_{j}\left|\right|$$r_{1}\left|\right|T_{3})$, and $SK=h(PID_{i}||PDID_{j}\left|\right|r_{1}||h(r_{2}\left|\right|$$R_{j}^{new}))$, and sends $\left\{PDID_{j},M_{5},M_{6},V_{3},T_{3}\right\}$ to the CSS through a public channel.
**Step** **4:** The CSS checks the validity of $T_{3}$. If it is valid, the CSS calculates $C_{j}^{new*}||MR_{j}^{new*}=M_{5}⊕h(PDID_{j}||MR_{j}\left|\right|a_{j}\left|\right|r_{1}\left|\right|T_{3})$, $h(r_{2}\left|\right|R_{j}^{new}{)}^{*}=M_{6}⊕h(PDID_{j}||MR_{j}^{new*}\left|\right|a_{j}\left|\right|T_{3})$, and $V_{3}^{*}=h(PDID_{j}\left|\right|C_{j}^{new*}||MR_{j}^{new*}||h(r_{2}\left|\right|R_{j}{new)}^{*}\left|\right|a_{j}\left|\right|r_{1}$$\left|\right|T_{3})$. Further, the CSS compares $V_{4}^{*}$ with $V_{4}$. After checking the equality, the CSS generates a timestamp $T_{4}$ and computes $PID_{i}^{new}=h(PID_{i}\left|\right|r_{1}\left|\right|T_{4})$, $M_{7}=h(r_{2}\left|\right|R_{j}^{new})⊕h(PID_{i}^{new}||PDID_{j}\left|\right|s_{i}\left|\right|$$RID_{i}\left|\right|T_{4})$, $V_{4}=h(PID_{i}^{new}||PDID_{j}\left|\right|s_{i}||RID_{i}||h(r_{2}\left|\right|R_{j}^{new})||T_{4})$, and $MR_{j}^{new^{\prime }}=MR_{j}^{new}⊕h(r_{j}\left|\right|X_{CSS})$. Then, the CSS transmits $\left\{M_{7},V_{4},T_{4}\right\}$ to $U_{i}$ and updates $\left\{C_{j}^{new},MR_{j}^{new^{\prime }},PID_{i}^{new}\right\}$.
**Step** **5:** $U_{i}$ verifies that $T_{4}$ is legitimate. If it is legitimate, $U_{i}$ computes $PID_{i}^{new*}=h(PID_{i}\left|\right|r_{1}\left|\right|T_{4})$, $h(r_{2}\left|\right|R_{j}^{new}{)}^{*}=M_{7}⊕h(PID_{i}^{new*}||PDID_{j}\left|\right|s_{i}||RID_{i}\left|\right|T_{4})$ and $V_{4}^{*}=h(PID_{i}^{new*}||PDID_{j}\left|\right|s_{i}||RID_{i}||h(r_{2}\left|\right|R_{j}^{new}{)}^{*}\left|\right|T_{4})$. Then, $U_{i}$ checks whether $V_{4}^{*}$ is equal to $V_{4}$. If they are equal, $U_{i}$ updates $PID_{i}^{new}$ and computes the session key $SK=h(PID_{i}||PDID_{j}\left|\right|r_{1}||h(r_{2}\left|\right|R_{j}^{new}))$.

### 6.5. Password Update Phase

**Step** **1:** $U_{i}$ inputs his/her identity $ID_{i}$ and password $PW_{i}$, and computes $e_{i}^{*}=f_{i}⊕h(ID_{i}||PW_{i})$ and $H_{i}^{*}=h(ID_{i}⊕e_{i}^{*}||PW_{i}⊕e_{i}^{*})$. Then, $U_{i}$ compares that $H_{i}^{*}$ and $H_{i}$ are equal or not. If they are equal, login is completed.
**Step** **2:** $U_{i}$ inserts new password $PW_{i}^{new}$. Then, $U_{i}$ calculates $f_{i}^{new}=e_{i}⊕h(ID_{i}||PW_{i}^{new})$, $H_{i}^{new}=h(ID_{i}⊕e_{i}||PW_{i}^{new}⊕e_{i})$, $RID_{i}^{\prime new}=RID_{i}⊕h(ID_{i}||PW_{i}^{new}\left|\right|e_{i})$, $PDID_{j}^{\prime new}=PDID_{j}⊕h(RID_{i}||ID_{i}||PW_{i}^{new})$, and $s_{i}^{\prime new}=s_{i}⊕h(RID_{i}||PW_{i}^{new}\left|\right|e_{i})$. Finally, $U_{i}$ stores $\left\{PID_{i},f_{i}^{new},H_{i}^{new},RID_{i}^{\prime new},PDID_{j}^{\prime new},s_{i}^{\prime new}\right\}$ to the database.

## 7. Security Analysis

Here, we discuss the approach to verifying the resilience of the proposed scheme. To formally validate the robustness of our scheme, we employ “BAN logic”, “RoR model”, “AVISPA”, and informal analysis. The results demonstrate that our scheme effectively resists various attacks while ensuring critical security requirements comprising mutual authentication, user anonymity, and untraceability. Further details are provided below.

### 7.1. BAN Logic

BAN logic is regarded as a standard analytical approach which is utilized to substantiate formally whether mutual authentication is achieved in AKA schemes. It has been extensively utilized by researchers to demonstrate the mutual authentication of various protocols. In this section, we first introduce the key notations and foundational rules of BAN logic. Subsequently, BAN logic analysis is applied to the proposed scheme. The primary BAN logic notations used in this study are summarized in Table 3. Further details of the analysis are as follows:

#### 7.1.1. Rules

The fundamental BAN logic rules utilized in this paper are outlined below.

Message meaning rule (MMR):

$$\frac{P_{1}|\equiv P_{1}\overset{K}{↔}P_{2},P_{1}◃{\left(M_{1}\right)}_{K}}{P_{1}\left|\equiv P_{2}\right|∼M_{1}}$$

Nonce verification rule (NVR):

$$\frac{P_{1}|\equiv \#M_{1},P_{1}\left|\equiv P_{2}\right|∼M_{1}}{P_{1}\left|\equiv P_{2}\right|\equiv M_{1}}$$

Jurisdiction rule (JR):

$$\frac{P_{1}|\equiv P_{2}\Rightarrow M_{1},P_{1}\left|\equiv P_{2}\right|\equiv M_{1}}{P_{1}|\equiv M_{1}}$$

Freshness rule (FR):

$$\frac{P_{1}|\equiv \#M_{1}}{P_{1}|\equiv \#\left(M_{1},M_{2}\right)}$$

Belief rule (BR):

$$\frac{P_{1}|\equiv \left(M_{1},M_{2}\right)}{P_{1}|\equiv M_{1}}$$

#### 7.1.2. Idealized Forms

Idealized forms are defined as below.

$Msg_{1}$:
$U_{i}\to CSS:{\left(PDID_{j},r_{1},T_{1}\right)}_{s_{i}}$

$Msg_{2}$:
$CSS\to D_{j}:{\left(PID_{i},r_{1},T_{2}\right)}_{a_{j}}$

$Msg_{3}$:
$D_{j}\to CSS:(h(r_{2}\left|\right|R_{j}^{new}{),T_{3})}_{a_{j}}$

$Msg_{4}$:
$CSS\to U_{i}:(h(r_{2}\left|\right|R_{j}^{new}{),T_{4})}_{s_{i}}$

#### 7.1.3. Goals

The security goals used to verify the guarantee of mutual authentication comprise the following:

**Goal 1**: $U_{i}|\equiv U_{i}\overset{SK}{↔}D_{j}$
**Goal 2**: $D_{j}|\equiv U_{i}\overset{SK}{↔}D_{j}$
**Goal 3**: $U_{i}\left|\equiv D_{j}\right|\equiv U_{i}\overset{SK}{↔}D_{j}$
**Goal 4**: $D_{j}\left|\equiv U_{i}\right|\equiv U_{i}\overset{SK}{↔}D_{j}$

#### 7.1.4. Assumptions

Assumptions are defined as follows:

$A_{1}$: $CSS|\equiv (U_{i}\overset{s_{i}}{↔}CSS)$
$A_{2}$: $CSS|\equiv \#(T_{1})$
$A_{3}$: $D_{j}|\equiv D_{j}\overset{a_{j}}{↔}CSS$
$A_{4}$: $D_{j}|\equiv \#\left(T_{2}\right)$
$A_{5}$: $CSS|\equiv (D_{j}\overset{a_{j}}{↔}CSS)$
$A_{6}$: $CSS|\equiv \#(T_{3})$
$A_{7}$: $U_{i}|\equiv \left(U_{i}\overset{s_{i}}{\leftarrow }CSS\right)$
$A_{8}$: $U_{i}|\equiv \#\left(T_{4}\right)$
$A_{9}$: $U_{i}|\equiv CSS\Rightarrow \left(U_{i}\overset{h(r_{2}||R_{j}^{new})}{⇌}D_{j}\right)$
$A_{10}$: $D_{j}|\equiv CSS\Rightarrow \left(U_{i}\overset{r_{1}}{⇌}D_{j}\right)$
$A_{11}$: $U_{i}|\equiv D_{j}\Rightarrow \left(U_{i}\overset{SK}{↔}D_{j}\right)$
$A_{12}$: $D_{j}|\equiv U_{i}\Rightarrow \left(U_{i}\overset{SK}{↔}D_{j}\right)$

#### 7.1.5. Proof

The procedure for the proof is described as follows:

Step 1: According to $Msg_{1}$, we can obtain $S_{1}$.

$$S_{1}:CSS◃{\left(PDID_{j},r_{1},T_{1}\right)}_{s_{i}}$$

Step 2: By applying $S_{1}$ and $A_{1}$ to the MMR, we can obtain $S_{2}$.

$$S_{2}:CSS\left|\equiv U_{i}\right|∼\left(PDID_{j},r_{1},T_{1}\right)$$

Step 3: By applying $A_{2}$ to the FR, we can obtain $S_{3}$.

$$S_{3}:CSS|\equiv \#\left(PDID_{j},r_{1},T_{1}\right)$$

Step 4: By applying $S_{2}$ and $S_{3}$ to the NVR, we can obtain $S_{4}$.

$$S_{4}:CSS\left|\equiv U_{i}\right|\equiv \left(PDID_{j},r_{1},T_{1}\right)$$

Step 5: According to $Msg_{2}$, we can obtain $S_{5}$.

$$S_{5}:D_{j}◃{\left(PID_{i},r_{1},T_{2}\right)}_{a_{j}}$$

Step 6: By applying $S_{5}$ and $A_{3}$ to the MMR, we can obtain $S_{6}$.

$$S_{6}:D_{j}\left|\equiv CSS\right|∼\left(PID_{i},r_{1},T_{2}\right)$$

Step 7: By applying $A_{4}$ to the FR, we can obtain $S_{7}$.

$$S_{7}:D_{j}|\equiv \#\left(PID_{i},r_{1},T_{2}\right)$$

Step 8: By applying $S_{6}$ and $S_{7}$ to the NVR, we can obtain $S_{8}$.

$$S_{8}:D_{j}\left|\equiv CSS\right|\equiv \left(PID_{i},r_{1},T_{2}\right)$$

Step 9: According to $Msg_{3}$, we can obtain $S_{9}$.

$$S_{9}:CSS◃(h(r_{2}\left|\right|R_{j}^{new}{),T_{3})}_{a_{j}}$$

Step 10: By applying $S_{9}$ and $A_{5}$ to the MMR, we can obtain $S_{10}$.

$$S_{10}:CSS|\equiv D_{j}|∼(h(r_{2}\left|\right|R_{j}^{new}),T_{3})$$

Step 11: By applying $A_{6}$ to the FR, we can obtain $S_{11}$.

$$S_{11}:CSS|\equiv \#(h(r_{2}\left|\right|R_{j}^{new}),T_{3})$$

Step 12: By applying $S_{10}$ and $S_{11}$ to the NVR, we can obtain $S_{12}$.

$$S_{12}:CSS|\equiv D_{j}|\equiv (h(r_{2}\left|\right|R_{j}^{new}),T_{3})$$

Step 13: According to $Msg_{4}$, we can obtain $S_{13}$.

$$S13:U_{i}◃(h(r_{2}\left|\right|R_{j}^{new}{),T_{4})}_{s_{i}}$$

Step 14: By applying $S_{13}$ and $A_{7}$ to the MMR, we can obtain $S_{14}$.

$$S_{14}:U_{i}|\equiv CSS|∼(h(r_{2}\left|\right|R_{j}^{new}),T_{4})$$

Step 15: By applying $A_{8}$ to the FR, we can obtain $S_{15}$.

$$S_{15}:U_{i}|\equiv \#(h(r_{2}\left|\right|R_{j}^{new}),T_{4})$$

Step 16: By applying $S_{14}$ and $S_{15}$ to the NVR, we can obtain $S_{16}$.

$$S_{16}:U_{i}|\equiv CSS|\equiv (h(r_{2}\left|\right|R_{j}^{new}),T_{4})$$

Step 17: We can obtain $S_{17}$ from $S_{12}$, $S_{16}$, and $A_{9}$ because the session key is $SK=h(PID_{i}||PDID_{j}\left|\right|r_{1}||h(r_{2}\left|\right|R_{j}^{new}))$.

$$S_{17}:U_{i}\left|\equiv D_{j}\right|\equiv \left(U_{i}\overset{SK}{↔}D_{j}\right)\;\left(\mathrm{Goal}3\right)$$

Step 18: By applying $S_{17}$ and $A_{11}$ to the JR, we can obtain $S_{18}$.

$$S_{18}:U_{i}|\equiv \left(U_{i}\overset{SK}{↔}D_{j}\right)\;\left(\mathrm{Goal}1\right)$$

Step 19: We can obtain $S_{19}$ from $S_{4}$, $S_{8}$, and $A_{10}$ because the session key is $SK=h(PID_{i}||PDID_{j}\left|\right|r_{1}||h(r_{2}\left|\right|R_{j}^{new}))$.

$$S_{17}:D_{j}\left|\equiv U_{i}\right|\equiv \left(U_{i}\overset{SK}{↔}D_{j}\right)\;\left(\mathrm{Goal}4\right)$$

Step 20: By applying $S_{19}$ and $A_{12}$ to the JR, we can obtain $S_{20}$.

$$S_{18}:D_{j}|\equiv \left(U_{i}\overset{SK}{↔}D_{j}\right)\;\left(\mathrm{Goal}2\right)$$

### 7.2. RoR Model

This section demonstrates the application of the RoR model to the proposed scheme. The RoR model is a well-known formal analysis that can verify whether an authentication protocol provides the semantic security of a session key [36,37,38]. Before explaining the application of the RoR model to the proposed scheme, we describe its basic concepts and notations. Under the RoR model, A executes queries that can attempt both active and passive attacks to reveal the session key. We describe the queries executed by A, as detailed below. We denote three participants—a user, a drone, and a CSS—as $P_{U}^{t_{1}}$, $P_{D}^{t_{2}}$, and $P_{CSS}^{t_{3}}$, respectively. The notation $t_{k}$ is defined as a participant instance of a user, a drone, and a CSS.

- *Execute ($P_{U}^{t_{1}}$, $P_{D}^{t_{2}}$, $P_{CSS}^{t_{3}}$)*: Using this query, A eavesdrops on messages transmitted over a public channel among $P_{U}^{t_{1}}$, $P_{D}^{t_{2}}$, and $P_{CSS}^{t_{3}}$.
- *Send ($P^{t}$, M)*: A message *M* can be transmitted to participant $P^{t}$ by A to receive a response message.
- *CorruptMD ($P_{U}^{t_{1}}$)*: This query denotes smart device stolen attacks. A can attempt to extract the secret parameters stored in a user’s smart device.
- *Test ($P^{t}$)*: Using this query, A determines if the speculative session key is a real session key or a random string. A fair coin *c* is flipped at the beginning of this query. A obtains c=1 when $P^{t}$ returns a real session key and c=0 when $P^{t}$ returns a random string. Otherwise, A receives a null. A is considered the winner of the game if A can judge whether the value output by $P^{t}$ is the session key or a random string.

**Theorem** **1.** *Consider A to attempt to compromise the proposed scheme within polynomial time. Let $Adv_{A}$ denote the advantage that A successfully distinguishes the session key from a random string. Consequently, we obtain the result of the advantage as follows:*

$$Adv_{A}\leq \frac{q_{h}^{2}}{\left|Hash\right|}+\frac{q_{p}^{2}}{\left|PUF\right|}+2max\left\{C\cdot q_{s}^{s^{\prime }},\frac{q_{s}}{2^{l}}\right\}$$

|PUF| *and |Hash| are defined as the output range of the PUF PUF(·) and the hash function H(·). Additionally, $q_{p}$ and $q_{h}$ denote the number of PUF and Hash queries executed by A, respectively.*

**Proof.**  The semantic security of the session key is verified as demonstrated in a series of games $G_{i}\left(i=0,1,2,3\right)$. $Pr[Succ_{i}]$ indicates the possibility that A correctly distinguishes *c* in $G_{i}$.

$Game_{0}$: At the start of the game, A selects a random bit *c*. Hence, we can obtain Equation (1).
  (1) $$Adv_{A}=\left|2Pr\left[Succ_{0}\right]-1\right|$$
$Game_{1}$: A attempts an eavesdropping attack by conducting an Execute query. Further, A runs Test queries to determine if the acquired value is a session key or not. A must know $PDID_{j}$, $r_{1}$, and $h(r_{2}R_{j}^{new})$ to acquire the session key $SK=h(PID_{i}PDID_{j}r_{1}h(r_{2}$$R_{j}^{new}))$. However, these values cannot be obtained by eavesdropping attacks. This means that A has no advantage to be gained through an Execute query. Therefore, the probability of A winning $G_{1}$ is equal to that of A winning $G_{0}$.
  (2) $$Pr\left[Succ_{1}\right]=Pr\left[Succ_{0}\right]$$
$Game_{2}$: In this game, A runs Send and Hash queries to expose the session key. The transmitted messages can be modified by A. However, A should find a hash collision to win the game because all transmitted messages are masked by a one-way function H(·). Therefore, the advantage that A can gain at the end of $G_{2}$ is obtained based on the birthday paradox.
  (3) $$|Pr\left[Succ_{2}\right]-Pr\left[Succ_{1}\right]|\leq \frac{q_{h}^{2}}{2|Hash|}$$
$Game_{3}$: Similar to $Game_{2}$, A runs Send and PUF queries. Due to security properties of the PUF described in Section 3.3, A cannot obtain an advantage after conducting $Game_{3}$.
  (4) $$|Pr\left[Succ_{3}\right]-Pr\left[Succ_{2}\right]|\leq \frac{q_{p}^{2}}{2|PUF|}$$
$Game_{4}$: In this game, A conducts CorruptMD queries to extract the secret parameters $\left\{PID_{i},f_{i},H_{i},RID_{i}^{\prime },PDID_{j}^{\prime },s_{i}^{\prime }\right\}$ from a user’s smart device, exploiting power analysis attacks. Further, A aims to derive the session key $SK=h(PID_{i}||PDID_{j}\left|\right|r_{1}||h(r_{2}\left|\right|R_{j}^{new}))$. However, each parameter consists of a user’s identity $ID_{i}$ and password $PW_{i}$. Therefore, A should guess the identity and password simultaneously. We can induce the following equation by adopting Zipf’s law [39]:
  (5) $$|Pr\left[Succ_{4}\right]-Pr\left[Succ_{3}\right]|\leq max\left\{C\cdot q_{s}^{s^{\prime }},\frac{q_{s}}{2^{l}}\right\}$$
  To win the game, A has to guess the bit *c* after finishing all games. Because A has no advantage in guessing *c*, we derive Equation (6).
  (6) $$Pr\left[Succ_{4}\right]=\frac{1}{2}$$
  Equation (7) is obtained from Equations (1) and (2).
  (7) $$\frac{1}{2}Adv_{A}=|Pr\left[Succ_{0}\right]-\frac{1}{2}|=|Pr\left[Succ_{1}\right]-\frac{1}{2}|$$
  Equation (8) is obtained based on Equations (6) and (7).
  (8) $$\frac{1}{2}Adv_{A}=\left|Pr\left[Succ_{1}\right]-Pr\left[Succ_{4}\right]\right|$$
  Equation (9) is obtained using the triangle inequality of Equation (8).
  (9) $$\begin{array}{c} \frac{1}{2}Adv_{A}=\left|Pr\left[Succ_{1}\right]-Pr\left[Succ_{4}\right]\right|\\ \;\;\leq |Pr\left[Succ_{1}\right]-Pr\left[Succ_{2}\right]|+|Pr\left[Succ_{2}\right]-Pr\left[Succ_{3}\right]|+|Pr\left[Succ_{3}\right]-Pr\left[Succ_{4}\right]|\\ \leq \frac{q_{h}^{2}}{2|Hash|}+\frac{q_{p}^{2}}{2|PUF|}+max\left\{C\cdot q_{s}^{s^{\prime }},\frac{q_{s}}{2^{l}}\right\} \end{array}$$
  Finally, the result is obtained by multiplying Equation (9) by 2.
  (10) $$Adv_{A}\leq \frac{q_{h}^{2}}{\left|Hash\right|}+\frac{q_{p}^{2}}{\left|PUF\right|}+2max\left\{C\cdot q_{s}^{s^{\prime }},\frac{q_{s}}{2^{l}}\right\}$$
  Consequently, Theorem 1 is verified. □

### 7.3. AVISPA Tool

This section presents the key data flow of AVISPA, highlighting the security verification of the proposed scheme. AVISPA is a widely accepted simulation tool used to prove whether a protocol is secure against replay attacks and MITM attacks. “High-Level Protocol Specification Language (HLPSL)” is a language used to execute a protocol in AVISPA based on a role. First, the HLPSL2IF translator converts the code written in HLPSL into an “Intermediate Format (IF)”. Then, AVISPA executes a simulation using four back-end models: “on-the-fly model checker (OFMC)”, “SAT-based model checker (SATMC)”, “constraint logic-based attack searcher (CL-AtSe)”, and “tree automata based on automatic approximations for the analysis of security protocols (TA4SP)”. If the IF is placed into the back-end by the translator, the back-end generates and summarizes the analysis result as an “output format (OF)”. An authentication protocol can resist MITM and replay attacks if the summary of OF represents “SAFE”.

**Figure 6:**
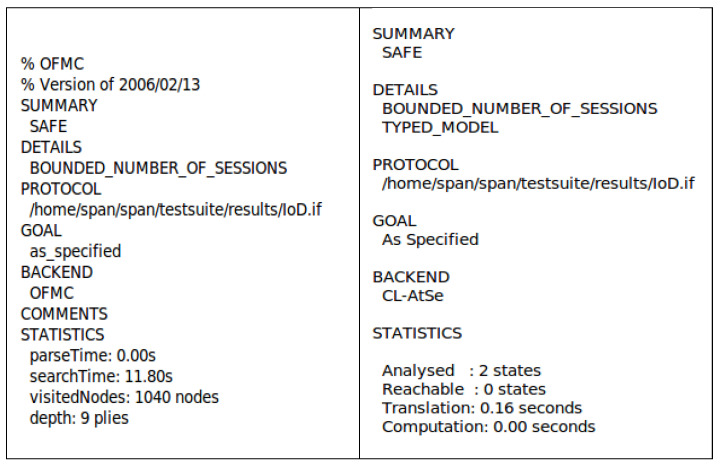
AVISPA simulation result under OFMC and CL-AtSe.

In this paper, we use two back-ends, “OFMC” and “CL-AtSe”, for the AVISPA simulation of the proposed scheme. There are three roles ($U_{i}$, $D_{j}$, and CSS) in HLPSL, and we describe session and environment roles within those three roles. The secrecy of the secret parameter and the appropriateness of mutual authentication are checked in each session. Figure 6 represents the simulation results, showing that the summaries present “SAFE” using the “OFMC” and “CL-AtSe” back-end models. Hence, replay and MITM attacks cannot be successfully performed by A.

### 7.4. Informal Analysis

We analyze the proposed scheme informally to demonstrate the robustness related to numerous attacks. We also confirm that the proposed scheme achieves security requirements, including mutual authentication, perfect forward secrecy, user anonymity and untraceability.

#### 7.4.1. Impersonation Attack

At the start of the AKA phase, $U_{i}$ transmits the request message $\left\{PID_{i},M_{1},M_{2},V_{1},T_{1}\right\}$ to the CSS first. A must compute the message to impersonate $U_{i}$. Under the adversary model, A can obtain the secret information $\left\{PID_{i},f_{i},H_{i},RID_{i}^{\prime },PDID_{j}^{\prime },s_{i}^{\prime }\right\}$ stored in the smart device of $U_{i}$. However, A cannot compute $\left\{PDID_{j},RID_{i},s_{i}\right\}$ because they are masked by $\left\{ID_{i},PW_{i},e_{i}\right\}$. A should guess $ID_{i}$ and $PW_{i}$ simultaneously to obtain $e_{i}=f_{i}⊕h(ID_{i}||PW_{i})$. It is computationally infeasible. As a result, our scheme prevents impersonation attacks.

#### 7.4.2. Stolen Verifier Attack

The CSS stores verification table $\left\{PID_{i},r_{i}\right\}$ in its database. According to the CK model, suppose that A steals the verification table. After obtaining the verification table, A can use the values $\left\{PID_{i},r_{i}\right\}$ to calculate the session key $SK=h(PID_{i}||PDID_{j}\left|\right|r_{1}||h(r_{2}\left|\right|R_{j}^{new}))$. However, A cannot obtain the secret parameter $\{PDID_{j},r_{1},h(r_{2}\left|\right|R_{j}^{new})\}$ without knowing the secret key $\left\{s_{i},a_{j}\right\}$. Although A has $\left\{PID_{i},r_{i}\right\}$, A cannot calculate $s_{i}$ and $a_{j}$. Thus, the proposed scheme can defend stolen verifier attacks.

#### 7.4.3. Ephemeral Secret Leakage Attack

A accesses to the ephemeral secrets $r_{1}$ and $r_{2}$, which are generated by $U_{i}$ and $D_{j}$ in the AKA phase. Further, A aims to acquire the session key $SK=h(PID_{i}||PDID_{j}\left|\right|r_{1}||h(r_{2}\left|\right|$$R_{j}^{new}))$. Even if A obtains the random secrets $r_{1}$ and $r_{2}$, A still does not know $PDID_{j}$ and $h(r_{2}||R_{j}^{new})$. A cannot acquire $PDID_{j}$ and $h(r_{2}||R_{j}^{new})$ without the secret key $a_{j}$ and $MR_{j}$, which are masked by the master key of the CSS and the PUF response of $D_{j}$. Hence, our scheme can resist against ESL attacks.

#### 7.4.4. Replay Attack

All the messages are hashed with timestamps during the AKA phase of the proposed scheme. Even if A intercepts a message transmitted through an open channel and tries to resend the message, A cannot reuse the message because each entity verifies the validity of the timestamp in every session. If a timestamp is not in a legitimate range, authentication will fail. Hence, the proposed scheme can defend replay attacks.

#### 7.4.5. Man-in-the-Middle Attack

After intercepting the message that $U_{i}$ or $D_{j}$ transmit to the CSS, A generates a random number and a timestamp, and attempts to modify the message to send another valid message. However, A cannot calculate the message $\left\{M_{1},M_{2},V_{1}\right\}$ because A does not know the secret parameters $RID_{i}$ and $s_{i}$ shared between $U_{i}$ and the CSS. Since $RID_{i}$ and $s_{i}$ are masked by the master key of the CSS and stored in a user’s smart device securely, A cannot obtain them. In a similar way, A also cannot compute the message $\left\{M_{5},M_{6},V_{3}\right\}$ due to the secrecy of $a_{j}$. Therefore, our scheme is resistant to MITM attacks.

#### 7.4.6. Privileged Insider Attack

The registration request message of $U_{i}$, $\left\{ID_{i}\right\}$ can be intercepted by a privileged adversary A. Then, A attempts to obtain the secret values $RID_{i}$ and $s_{i}$ using $ID_{i}$. Even if A obtains $ID_{i}$, A cannot calculate $RID_{i}$ and $s_{i}$ because they are hashed with the master key of the CSS $X_{CSS}$. Each of the parameters necessary for calculating the session key $SK=h(PID_{i}||PDID_{j}||r_{1}||$$h(r_{2}||R_{j}^{new}))$ are encrypted with $RID_{i}$ and $s_{i}$. Therefore, A cannot successfully defend against privileged insider attacks.

#### 7.4.7. Drone Capture Attack

A can attempt to derive the session key $SK=h(PID_{i}||PDID_{j}\left|\right|r_{1}||h(r_{2}\left|\right|R_{j}^{new}))$ after A intercepts a drone $D_{j}$ and extracts the information $\left\{PDID_{j},b_{j}\right\}$. However, A cannot obtain the session key due to the secure property of the PUF. A must obtain $a_{j}$ and $MR_{j}$ to calculate the session key. However, these values are masked by the PUF response $R_{j}$. It is impossible to compute $R_{j}=PUF\left(C_{j}\right)$ for A. Additionally, the proposed scheme updates $R_{j}$ to $R_{j}^{new}$ in every session. Thus, our scheme is robust to drone capture attacks.

#### 7.4.8. Mutual Authentication

$U_{i}$, $D_{j}$ and the CSS verify the legitimacy of the message during the AKA phase. The CSS and $U_{i}$ authenticate each other by checking that $V_{1}^{*}$ is equal to $V_{1}$ and $V_{4}^{*}$ is equal to $V_{4}$. Similarly, the CSS and $D_{j}$ authenticate each other by verifying whether $V_{2}^{*}$ and $V_{2}$ are equal or not, and whether $V_{3}^{*}$ and $V_{3}$ are equal or not. If the values are not identical, the authentication process is terminated. $U_{i}$ and $D_{j}$ mutually authenticate each other and share a session key through CSS arbitration. Hence, mutual authentication is preserved in the proposed scheme.

#### 7.4.9. User Anonymity and Untraceability

The identity of $U_{i}$ is transmitted through a secure channel one time when $U_{i}$ registers itself to the CSS. Then, the CSS calculates a user’s pseudo-identity $PID_{i}$ and sends it to $U_{i}$. In the AKA phase, only $PID_{i}$ is used during communication. After terminating the key agreement, $U_{i}$ and the CSS update $PID_{i}$ to new a pseudo-identity $PID_{i}^{new}$. Thus, our scheme provides user anonymity and untraceability.

#### 7.4.10. Perfect Forward Secrecy

According to the adversarial assumptions described in Section 3.2, A can obtain the mater key of the CSS $X_{CSS}$. A uses $X_{CSS}$ to calculate the session key $SK=h(PID_{i}||PDID_{j}||r_{1}||$$h(r_{2}||R_{j}^{new}))$. However, $r_{1}$ and $h(r_{2}||R_{j}^{new})$ are transmitted while being encrypted by secret keys $s_{i}$ and $a_{j}$. Even if A gains $X_{CSS}$, A cannot obtain $s_{i}=h(RID_{i}\left|\right|r_{i}\left|\right|X_{CSS})$ and $a_{j}=h(PDID_{j}\left|\right|r_{j}\left|\right|X_{CSS})$. As a result, the proposed scheme guarantees perfect forward secrecy.

## 8. Performance Analysis

We present a performance comparison between the proposed scheme and related schemes. We estimate “security properties”, “computational cost”, “communication cost” and “energy consumption” of the proposed scheme and show that our scheme offers enhanced robustness and efficiency compared to others.

### 8.1. Security Properties

We examine the proposed scheme and comparable other schemes [15,16,17,18,19] regarding security features. We contemplate the following security functionalities: $S_{1}$: “resistance to impersonation attack”, $S_{2}$: “resistance to stolen verifier attack”, $S_{3}$: “resistance to ESL attack”, $S_{4}$: “resistance to replay attack”, $S_{5}$: “resistance to MITM attack”, $S_{6}$: “resistance to privileged insider attack”, $S_{7}$: “resistance to drone capture attack”, $S_{8}$: “ensuring user anonymity and untraceability”, $S_{9}$: “ensuring perfect forward secrecy”, $S_{10}$: “performing BAN logic”, $S_{11}$: “performing RoR model”, and $S_{12}$: “performing AVISPA”. We summarize the comparative analysis in Table 4. The proposed scheme achieves abundant security properties that are necessary for IoD communication.

### 8.2. Computational Costs

This section focuses on analyzing the computational cost of the proposed scheme compared to other related works [15,16,17,18,19]. We quote the work using ubuntu 12.04.1 LTS 32-bit operating system, 2048 MB of RAM, and Intel Pentium Dual CPU E2200 2.20 GHz processor [15]. $T_{HECC}$, $T_{fe}$, $T_{sym}$, $T_{ag}$, $T_{PUF}$ and $T_{h}$ represent HECC divisor multiplication, fuzzy extractor function, symmetric encryption/decryption, AEGIS (AEAD scheme), PUF, and hash function. Table 5 depicts the execution time of the operations. We disregard the time cost of XOR and concentration operations, have extremely low computation costs [40]. In the proposed scheme, a user requires $12T_{h}$, a CSS requires $17T_{h}$, and a drone requires $2T_{PUF}+13T_{h}$. Therefore, the total time overhead incurred by each entity is $2T_{PUF}+42T_{h}$. Similarly, we also compute the computational costs of the related schemes and compare them with our scheme. We represent the result of the comparison in Table 6. Although the proposed scheme incurs a slightly higher computation time than [16,19], the proposed scheme provides enhanced security. Zhang et al.’s scheme [16] is vulnerable to replay and privileged insider attacks, as outlined in Table 4. In the IoD environment, A can illegally control the drones to carry out malicious operations by resending intercepted authentication messages. A can also cause malfunctions or disruptions in drone operations to manipulate the IoD system through privileged insider attacks. Therefore, the security drawbacks of their scheme are fatal in IoD environments. Additionally, Sharma et al.’s scheme cannot withstand impersonation, stolen verifier, and ESL attacks, as demonstrated above. Therefore, our scheme has an efficient balance in terms of time cost and security.

### 8.3. Communication Costs

**Figure 7:**
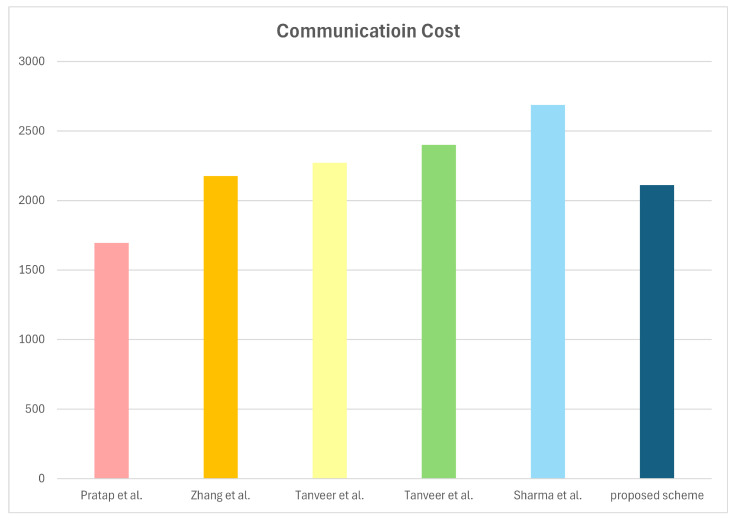
Communication costs [15,16,17,18,19].

We conduct a comparison of communication costs between our scheme and associated schemes [15,16,17,18,19]. In this paper, we consider the size of the PUF response, authentication parameter, hash function output, random number, identity, AES block, MC, HECC divisor, PUF challenge, and timestamp as 320 bits, 256 bits, 160 bits, 160 bits, 160 bits, 128 bits, 128 bits, 80 bits 32 bits, and 32 bits, respectively. In the proposed scheme, all entities transmit four messages, including $Msg1=\{PID_{i},M_{1},M_{2},V_{1},T_{1}\}$, $Msg2=\{M_{3},M_{4},V_{2},T_{2}\}$, $Msg3=\{M_{5},M_{6},V_{3},T_{3}\}$, and $Msg4=\{M_{7},V_{4},T_{4}\}$. The communication costs of the messages are 160+160+160+160+32=672bits, (160+32)+160+160+32=544bits, (160+32)+160+160+32=544bits, and 160+160+32=352bits. Therefore, the total number of bits is 672+672+512+352=2112bits. We also compute the communication costs of relevant approaches. Table 7 and Figure 7 represent the communication costs of the proposed scheme and relevant approaches. The comparative analysis indicates a high communication efficiency of the proposed scheme.

### 8.4. Energy Consumption

**Figure 8:**
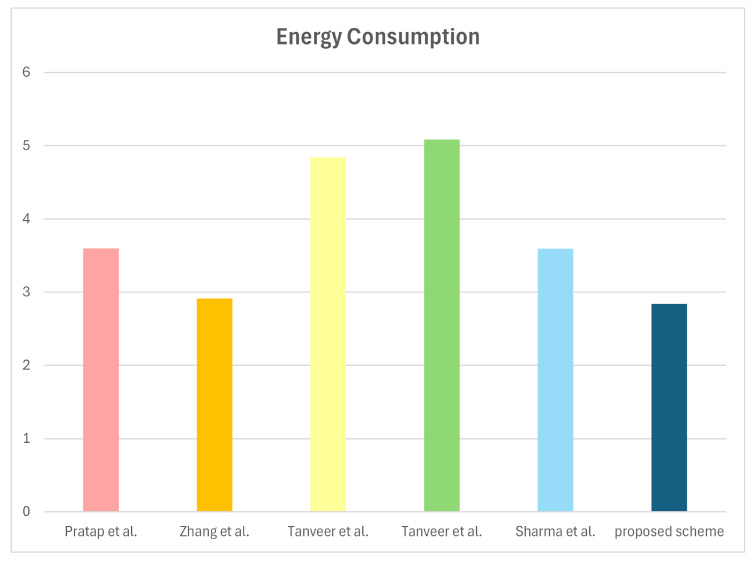
Energy consumption [15,16,17,18,19].

Energy consumption can be calculated with $E=E_{comp}+E_{comm}$. Based on the equation, we estimate the energy overhead of our scheme with relevant schemes [15,16,17,18,19]. $E_{comp}$ denotes the energy consumption during computation and $E_{comm}$ denotes the energy consumption during communication [41]. According to test works conducted in [42] and the execution time in Table 5 measured by the equipment described in Section 8.2, we can compute the energy consumption for the “HECC divisor”, “fuzzy extractor”, “symmetric encryption/decryption”, “AEGIS”, “PUF”, and “hash function” to be $E_{HECC}=0.5\;V\times 0.4\;A\times 1.113\;\mathrm{ms}=0.2226\;\mathrm{mJ}$, $E_{fe}=0.4452\;\mathrm{mJ}$, $E_{sym}=0.00092\;\mathrm{mJ}$, $E_{ag}=0.083\;\mathrm{mJ}$, $E_{PUF}=0.0108$ and $E_{h}=0.00046\;\mathrm{mJ}$, respectively. Additionally, according to [42], communication energy consumption can be calculated as $E_{comm}=n_{s}E_{s}+n_{r}E_{r}$, where $n_{s}$ denotes the number of bytes sent by the communication entity and $n_{r}$ denotes the number of bytes received by the communication entity. Further, we assume that energy costs of sending and receiving message are $E_{s}\approx 5.9\;\mu J$, and $E_{r}\approx 4.7\;\mu J$ [43]. Therefore, the energy consumption of the proposed protocol during computation and communication are calculated to be $E_{comp}=2E_{PUF}+42E_{h}=0.04092\;\mathrm{mJ}$, and $E_{comm}=264E_{s}+264E_{r}=2.7984\;\mathrm{mJ}$. Consequently, the proposed scheme incurs the energy consumption of 2.83932mJ. Comparison of energy consumption with associated schemes is depicted in Table 8 and Figure 8. The proposed scheme demonstrates more sustainable energy consumption compared to other related schemes.

## 9. Conclusions

In this paper, we provided the overview of Sharma et al.’s AKA scheme and conducted a security analysis of it. We verified that their scheme is susceptible against user impersonation, stolen verifier, and ESL attacks. Then, we proposed a lightweight and secure AKA scheme for the IoD to rectify the vulnerabilities of Sharma et al.’s scheme. Fundamental necessities required for IoD communication are guaranteed through our scheme. The proposed scheme is robust to numerous adversarial attacks comprising impersonation, stolen verifier, ESL, MITM, replay, drone physical attacks. We consider the resilience of the scheme as well as the resource limitations of drones. The proposed scheme utilizes lightweight operations such as the hash function, XOR operation, and PUF. We verified the secureness of our scheme with informal analysis. We also demonstrated the security of our scheme by formally employing “BAN logic”, “RoR model”, and “AVISPA”. We represented the efficiency of the proposed scheme, comparing it with other associated schemes. The result of our comparison showed that our scheme is highly cost-effective with robustness regarding its computational cost, communication cost, and energy consumption. Therefore, the proposed scheme allows the IoD to provide improved services. It also involves a higher number of message exchanges in the authentication phase compared with other related schemes. However, the overall communication costs remain efficient because each message has a lower cost in comparison to the compared schemes. Moreover, the proposed scheme considers a wide range of security properties and provides robust protection against various security threats. In our future work, we will implement the proposed scheme, optimizing and confirming its scalability and energy efficiency in a practical large-scale IoD environment.

## Figures and Tables

**Table 1:** Summary of the proposed scheme and related schemes.

Year	Scheme	Contributions	Limitations
2024	[15]	Proposed a mutual AKA scheme for the IoD environment Using HECC	Cannot prevent drone capture attacks Large computation cost
2024	[16]	Proposed a lightweight AKA scheme for the IoD environment Considered computation costs for drones Using PUF and hash functions	Cannot prevent replay and privileged insider attacks
2024	[17]	Proposed a biometric-based AKA scheme for the IoD environment Using PUF and symmetric encryption	Large computation cost Does not consider various security properties
2024	[18]	Proposed a PUF-based AKA scheme for the IoD environment Using PUF and AEGIS	Large computation cost Does not consider various security properties
2023	[19]	Introduced a lightweight AKA scheme for the IoD environment Using PUF to prevent physical attacks on the drones	Cannot prevent impersonation, stolen verifier, ESL attacks Cannot ensure user anonymity and untraceability
-	Proposed	Propose a lightweight AKA scheme between user and drone for the IoD environment Address the security vulnerabilities of Sharma et al.’s scheme using PUF and hash function Consider resource limitations of the drones	

**Table 2:** Notations.

Notations	Descriptions
CSS	Control server station
$U_{i}$	*i*-th user
$D_{j}$	*j*-th drone
$X_{CSS}$	Master key of CSS
$ID_{i}$	Identity of $U_{i}$
$DID_{j}$	Identity of $D_{j}$
$PDID_{j}$	Pseudo identity of $D_{j}$
⊕	Exclusive-OR operation
*h*	One-way hash function
$T_{i}$	Timestamp
SK	Session key

**Table 3:** Notations in BAN logic.

Notations	Descriptions
$P_{1},P_{2}$	Principals
$M_{1},M_{2}$	Statements
SK	Session key
$P_{1}\overset{K}{↔}P_{2}$	$P_{1}$ and $P_{2}$ share the key *K*
$P_{1}|\equiv M_{1}$	$P_{1}$ believes $M_{1}$
$\#M_{1}$	$M_{1}$ is fresh
$P_{1}|∼M_{1}$	$P_{1}$ said $M_{1}$
$P_{1}\Rightarrow M_{1}$	$P_{1}$ controls $M_{1}$
$P_{1}◃M_{1}$	$P_{1}$ receives $M_{1}$
$P_{1}\overset{K}{⇌}P_{2}$	K is only known to trusted principals $P_{1}$ and $P_{2}$
${\left\{M_{1}\right\}}_{K}$	$M_{1}$ is masked by *K*

**Table 4:** Security properties.

Security Features	[15]	[16]	[17]	[18]	[19]	Proposed
$S_{1}$	∘	∘	∘	∘	×	∘
$S_{2}$	−	−	−	−	×	∘
$S_{3}$	∘	−	∘	∘	×	∘
$S_{4}$	∘	×	∘	∘	∘	∘
$S_{5}$	∘	−	∘	∘	∘	∘
$S_{6}$	−	×	∘	−	∘	∘
$S_{7}$	×	∘	∘	∘	∘	∘
$S_{8}$	∘	∘	∘	∘	×	∘
$S_{9}$	−	∘	−	−	−	∘
$S_{10}$	−	−	∘	−	−	∘
$S_{11}$	∘	∘	∘	∘	∘	∘
$S_{12}$	−	∘	−	−	∘	∘

∘: “Guarantee the security property.” ×: “Do not guarantee the security property.” −: “Not considered.”

**Table 5:** Execution time.

$T_{\mathrm{HECC}}$	$T_{\mathrm{fe}}$	$T_{\mathrm{sym}}$	$T_{\mathrm{ag}}$	$T_{\mathrm{PUF}}$	$T_{h}$
1.113 ms	2.226 ms	0.0046 ms	0.415 ms	0.054 ms	0.0023 ms

**Table 6:** Computational costs.

Protocol	User	Server	Drone	Total Cost (ms)
Pratap et al. [15]	$2T_{HECC}+T_{fe}+9T_{h}$	$4T_{h}$	$2T_{HECC}+4T_{h}$	6.7171
Zhang et al. [16]	$8T_{h}$	$6T_{h}$	$2T_{PUF}+6T_{h}$	0.154
Tanveer et al. [17]	$5T_{sym}+2T_{fe}+T_{PUF}+7T_{h}$	$5T_{sym}+T_{fe}+T_{PUF}+3T_{h}$	$3T_{sym}+T_{fe}+T_{PUF}+5T_{h}$	9.1603
Tanveer et al. [18]	$5T_{ag}+T_{fe}+4T_{h}$	$5T_{ag}+6T_{h}$	$2T_{ag}+T_{fe}+T_{PUF}+3T_{h}$	9.5159
Sharma et al. [19]	$8T_{h}$	$10T_{h}$	$2T_{PUF}+6T_{h}$	0.1632
proposed scheme	$12T_{h}$	$17T_{h}$	$2T_{PUF}+13T_{h}$	0.2046

**Table 7:** Communication costs.

Protocol	Communication Cost (bits)
Pratap et al. [15]	1696
Zhang et al. [16]	2176
Tanveer et al. [17]	2272
Tanveer et al. [18]	2400
Sharma et al. [19]	2688
proposed scheme	2112

**Table 8:** Energy consumption.

Protocol	Energy Consumption (mJ)
Pratap et al. [15]	3.59844
Zhang et al. [16]	2.914
Tanveer et al. [17]	4.84246
Tanveer et al. [18]	5.08318
Sharma et al. [19]	3.59424
proposed scheme	2.83932

## Data Availability

Data are contained within this article.
